# Memory Th17 cell-mediated protection against lethal secondary pneumococcal pneumonia following influenza infection

**DOI:** 10.1128/mbio.00519-23

**Published:** 2023-05-24

**Authors:** Yong Li, Ying Yang, Dafan Chen, Yan Wang, Xinyun Zhang, Wenchao Li, Shengsen Chen, Sandy M. Wong, Mengwen Shen, Brian J. Akerley, Hao Shen

**Affiliations:** 1 Department of Microbiology, University of Pennsylvania Perelman School of Medicine, Philadelphia, Pennsylvania, USA; 2 Shanghai Institute of Immunology, Shanghai Jiaotong University, Shanghai, China; 3 Shanghai Sixth People’s Hospital Affiliated to Shanghai Jiao Tong University School of Medicine, Shanghai, China; 4 Department of Infectious Diseases, Shanghai Key Laboratory of Infectious Diseases and Biosafety Emergency Response, National Medical Center for Infectious Diseases, Huashan Hospital, Fudan University, Shanghai, China; 5 Department of Endoscopy, Cancer Hospital of the University of Chinese Academy of Sciences (Zhejiang Cancer Hospital), Institute of Cancer and Basic Medicine (IBMC), Chinese Academy of Sciences, Hangzhou, Zhejiang, China; 6 Department of Microbiology and Immunology, University of Mississippi Medical Center, Jackson, Mississippi, USA; 7 Department of Emergency Medical, Yueyang Hospital of Integrated Traditional Chinese and Western Medicine Affiliated to Shanghai University of Traditional Chinese Medicine, Shanghai, China; 8 Department of Cell and Molecular Biology, Center for Immunology and Microbial Research, University of Mississippi Medical Center, Jackson, Mississippi, USA; UCLA School of Medicine, Los Angeles, California, USA

**Keywords:** influenza A virus, *Streptococcus pneumoniae*, coinfection, Th17 responses, cross-protection

## Abstract

*Streptococcus pneumoniae* (*Sp*) frequently causes secondary pneumonia after influenza A virus (IAV) infection, leading to high morbidity and mortality worldwide. Concomitant pneumococcal and influenza vaccination improves protection against coinfection but does not always yield complete protection. Impaired innate and adaptive immune responses have been associated with attenuated bacterial clearance in influenza virus-infected hosts. In this study, we showed that preceding low-dose IAV infection caused persistent *Sp* infection and suppression of bacteria-specific T-helper type 17 (Th17) responses in mice. Prior *Sp* infection protected against subsequent IAV/*Sp* coinfection by improving bacterial clearance and rescuing bacteria-specific Th17 responses in the lungs. Furthermore, blockade of IL-17A by anti-IL-17A antibodies abrogated the protective effect of *Sp* preinfection. Importantly, memory Th17 responses induced by *Sp* preinfection overcame viral-driven Th17 inhibition and provided cross-protection against different *Sp* serotypes following coinfection with IAV. These results indicate that bacteria-specific Th17 memory cells play a key role in providing protection against IAV/*Sp* coinfection in a serotype-independent manner and suggest that a Th17-based vaccine would have excellent potential to mitigate disease caused by coinfection.

**IMPORTANCE:**

*Streptococcus pneumoniae* (*Sp*) frequently causes secondary bacterial pneumonia after influenza A virus (IAV) infection, leading to increased morbidity and mortality worldwide. Current pneumococcal vaccines induce highly strain-specific antibody responses and provide limited protection against IAV/*Sp* coinfection. Th17 responses are broadly protective against *Sp* single infection, but whether the Th17 response, which is dramatically impaired by IAV infection in naïve mice, might be effective in immunization-induced protection against pneumonia caused by coinfection is not known. In this study, we have revealed that *Sp*-specific memory Th17 cells rescue IAV-driven inhibition and provide cross-protection against subsequent lethal coinfection with IAV and different *Sp* serotypes. These results indicate that a Th17-based vaccine would have excellent potential to mitigate disease caused by IAV/*Sp* coinfection.

## INTRODUCTION

Influenza A virus (IAV) is a major cause of epidemic and pandemic flu. The majority of patients with IAV infection have mild, self-limited illnesses, although the frequency of severe and fatal cases is increased during pandemics (1). Most severe disease and mortality during influenza pandemics are associated with secondary bacterial infection (2, 3). *Streptococcus pneumoniae* (*Sp*) is the most common bacterium found in secondary bacterial infections during influenza epidemics and pandemics (4, 5). *Sp* is a gram-positive bacterium causing upper respiratory tract infection, community-acquired pneumonia, and invasive disease including bacteremia and meningitis (6 - 8). In addition, secondary bacterial infection by *Sp* following initial influenza infection is specifically associated with high mortality rates, particularly during influenza pandemics (8).

Secondary bacterial infection following IAV remains a significant health burden worldwide, despite available commercial vaccines for both IAV and *Sp*. Concomitant pneumococcal and influenza vaccination improves protection against coinfection but does not always yield complete protection (9 - 11). In addition, the live attenuated influenza vaccine may promote off-target bacterial colonization, which would increase pneumococcal transmission and prevalence, contributing to excess pneumococcal invasive disease (12). Recently, it has been shown that persistence of bacterial carriage and transmigration to the middle ear were increased in live attenuated influenza virus-immunized mice (13, 14). Unlike flu vaccination, pneumococcal conjugate and polysaccharide vaccines (PCVs and PPSVs), such as PCV7, PCV13, and PPSV23, have only a moderate effect on bacterial clearance in murine models of IAV/*Sp* coinfection (11, 15, 16). In addition, the current PCVs are based on capsular polysaccharides conjugated to a protein carrier, and induce highly serotype-specific antibody responses against a limited set of strains (17, 18). However, to date, over 90 distinct serotypes of *Sp* have been identified that are characterized by structural and compositional variations in their capsules (19). Thus, there is an urgent, global need for an alternative pneumococcal vaccine that can provide broad protection against circulating strains of diverse serotypes and coinfection.

Several mechanisms accounting for severe morbidity and mortality following coinfections have recently been elucidated, including pathogen synergy and impaired antibacterial immune responses (20, 21). IAV can damage the epithelial lining of airways, which decreases mechanical clearance of bacteria and allows bacteria carried in the nasopharynx to spread to sites of disease including the lung and middle ear, or to become invasive and disseminate into the blood (22). IAV infection can also promote the adherence of *Sp* to lung epithelial cells through viral components such as neuraminidase (23, 24). In addition, type I and type II interferons (IFNs) induced by preceding influenza infection impair immunity against subsequent bacterial pneumoniae by disrupting phagocyte function, decreasing antimicrobial peptide production, and inhibiting lymphocyte-mediated responses such as IL-17 production in mice (25 - 28). As evidence, knockouts of type I IFNs and type II IFNs can rescue the innate immune response and provide protection against coinfection (24, 29, 30). Nevertheless, the role of adaptive immune responses against bacteria in coinfection is not well understood, and this knowledge is vital for vaccine development.

T-helper type 17 (Th17) cells are abundantly present in the lung and contribute to clearance of primary infections by extracellular bacteria including *Sp* (31). We and others have also shown that bacteria-specific Th17 cells induced by immunization provide more broad protection than the humoral response which is the main form of immunity induced by PCV (32, 33). Importantly, bacteria-specific memory Th17 cells induced by bacterial immunization protect against subsequent (heterologous) challenge with pneumococcal strains of different serotypes in a single bacterial infection model (32). Due to the serotype-independent protection induced by Th17 cells, current studies are focused on seeking Th17-based vaccines for prevention of *Sp* infection (34, 35). Although Th17 cells have been shown to confer broad protection in pneumonia caused by bacterial infection alone, antibacterial Th17 responses are inhibited by preceding influenza infection (36). In addition, Th17 cells exhibit a high level of plasticity and are capable of transdifferentiating to other Th subsets such as Th1 and Th2 in the context of other Th-driving environments such as flu infection (37). Therefore, it is not known if preexisting memory Th17 cells can overcome the pronounced impairment of anti-pneumococcal Th17 responses caused by IAV, leading to effective protection against coinfection in the lung. Influenza infection may not only impair primary Th17 response, but also the recall response by memory Th17 cells, resulting in decreased protection against secondary bacterial infection. In this study, we refined approaches to induce coinfection in mice with prior low-dose flu infection, more closely mimicking natural acquisition of influenza infection in humans. We found that prior low-dose flu infection increased susceptibility and mortality to secondary pneumococcal infection, and the number of *Sp*-specific Th17 cells was decreased in coinfection compared with that of *Sp* single infection. We further investigated the protection against coinfection provided by prior lung *Sp* infection and its immune mechanism. Our results showed that prior lung *Sp* infection conferred protection against coinfection by a memory Th17 response that was dependent on IL-17A. In addition, *Sp* preinfection induced cross-protection against coinfection with *Sp* of different serotypes.

## MATERIALS AND METHODS

### Animals

Female C57BL/6 mice (6–8 weeks old) were purchased from the National Cancer Institute (Fredericksburg, MD). Animal experiments were performed in accordance with The University of Pennsylvania Institutional Animal Care and Use Committee protocols.

### Pathogens and infections

*Sp* strain TIGR4 (serotype 4) and P1121 (serotype 23F) were grown in tryptic soy broth or on agar plates and used as described previously (32). Mice were infected and immunized as described previously (32). Briefly, mice were anesthetized by intraperitoneal injections with 100 µL ketamine/xylazine (100 mg/3.8 mg kg^−1^) and inoculated with 30 µL of *Sp* suspensions (10^6^ CFU) in phosphate-buffered saline (PBS) intranasally. *Haemophilus influenzae* NT127 was grown in brain heart infusion (BHI) broth supplemented with 2.0% (v/v) fildes enrichment and 10 µg/mL NAD (sBHI), or on sBHI agar plates.

Influenza A/Puerto Rico/8/34 (PR8; H1N1) virus strain was propagated in specific pathogen-free fertile chicken eggs and used to infect mice as previously described (38). Mice were anesthetized with intraperitoneally injected 100 µL ketamine/xylazine (100 mg/3.8 mg kg^−1^) before intranasal infection with 30 µL of PBS containing 50–200 TCID_50_ of PR8.

For coinfection, mice were intranasally infected with PR8 at dose of 50 TICD_50_ and 5 days later challenged with sublethal dose (10^6^ CFU) of *Sp* T4. Infected mice were weighed daily and those that lost more than 30% of their initial body weight were euthanized according to institutional guidelines. In survival curves, these moribund mice were considered nonsurvivors. Bacterial loads in bronchoalveolar lavage fluid (BALF), lung homogenates and blood were determined as described previously (32). The limit of detection for bacteria in lavage or lung homogenate was 10 CFU mL^−1^. Viral loads in lung homogenates were determined by quantitative real-time reverse transcription polymerase chain reaction (RT-PCR) on lung RNA for polymerase (PA) mRNA of the PR8 influenza virus. The housekeeping gene HPRT of mouse was used to normalize levels of IAV PA mRNA expression (39, 40).

### Heart rate, breath rate, and pulse oximetry measurements

Heart rate, breath rate, and arterial O_2_ saturation of individual mice was measured using noninvasive, real-time pulse oximetry (MouseOx, STARR Life Sciences, Oakmont) on conscious (nonanesthetized) mice using a collar clip after infection.

### Flow cytometry and enzyme-linked immunosorbent assay

Lymphocytes from the lungs were isolated and stained as described previously (32, 41). For intracellular staining, cells were stimulated with heat-killed bacteria (65℃ for 30 min) at the indicated multiplicity of infection for 16 h at 37°C with Golgi Plug/Stop added for the last 5 h, and then stained as described (32, 41). Cells were stimulated ex-vivo with PMA (50  ng/mL) and ionomycin (1 µg/mL) for 5 h at 37°C in the presence of BD GolgiStop to detect the total T cell responses. Enzyme-linked immunosorbent assay (ELISA) kits were used for detection of IL-17A and IFN-γ from BALF following the manufacturer’s protocol (BioLegend).

The bacteria-specific antibody response was measured by ELISA as previously described (41). Briefly, 96-well ELISA plates were coated with heat-killed bacteria. Wells were blocked with 2% nonfat milk in PBS and serial dilutions of BALF were applied to the wells. Plates were incubated with sera for 1 h at 37°C. Antibodies were detected with goat antimouse IgG-HRP (H+L). For detection, the TMB peroxidase substrate reagent set was used according to the manufacturer’s instructions (BioLegend). Optical densities were read at 450 nm with a microplate reader (Molecular Devices SpectraMax 190 Microplate Reader). Bound antibody in BALF was detected with goat antimouse IgG antibody (Invitrogen).

### *In vivo* IL-17 neutralization

*In vivo* IL-17 neutralization was achieved as previously described with slight modifications (32). Mice received 180 µg intraperitoneally of anti-IL-17 (clone 17F3) on days −1, 0, and 1 and 20 µg intranasally on day 0 following *Sp* challenge. Neutralizing efficiency was verified using ELISA for BALF on day 1 after neutralizing antibody injection.

### Statistical analyses

Unpaired, one-tailed, Student’s *t* tests were used to calculate statistical significance between the two groups and one-way analysis of variance was used for comparison of multiple groups followed by Bonferroni correction unless stated otherwise. *P* values are depicted as follows: ****P < 0.0001; ***P < 0.001; **P < 0.01; *P < 0.05, and ^NS^*P* > 0.05. A *P*-value ≤0.05 was considered significant.

## RESULTS

### Prior low-dose flu infection increases susceptibility and mortality to secondary bacterial infection

**Fig 1:**
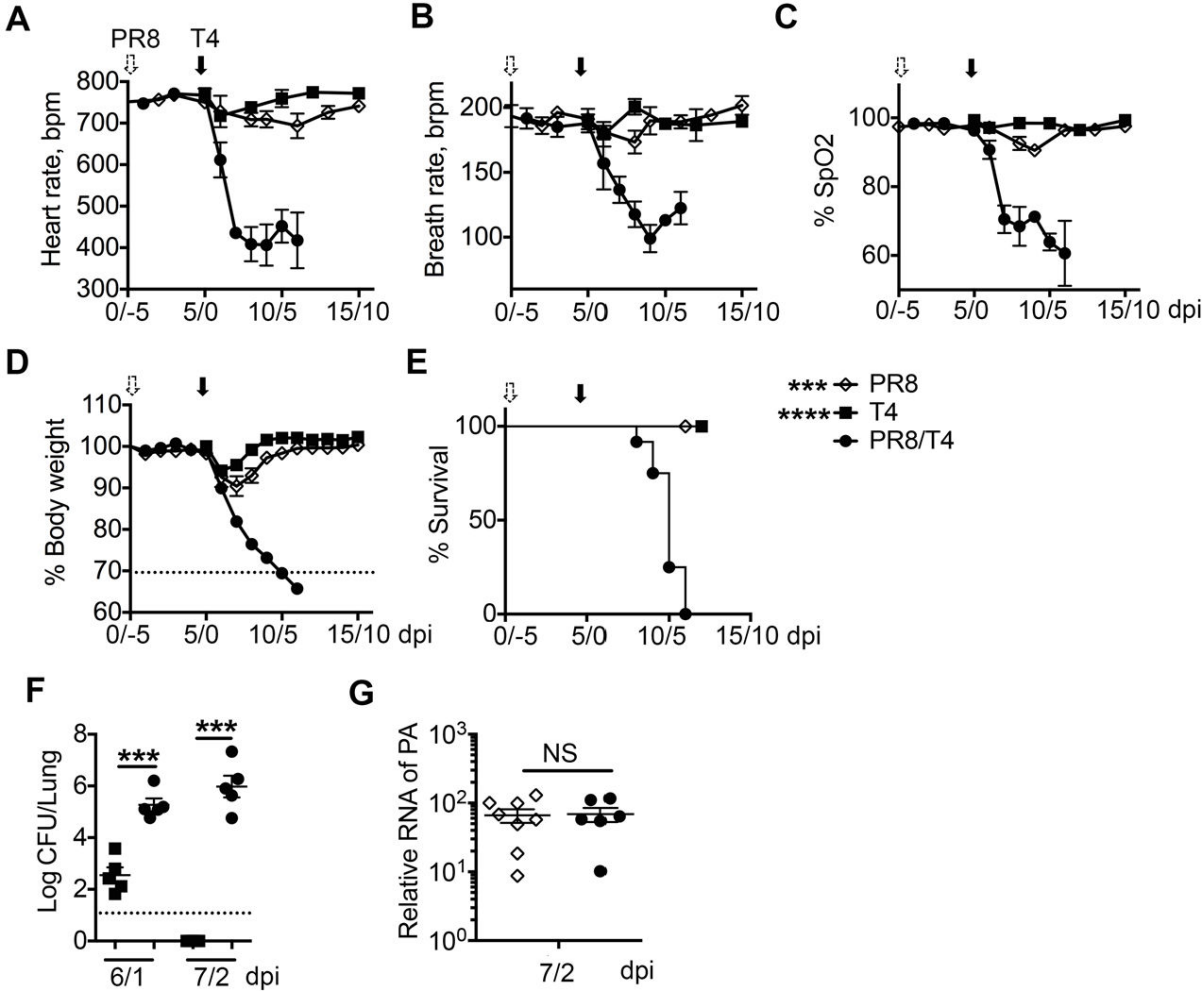
Low-dose flu infection increases susceptibility and mortality during secondary bacterial infection. Mice were infected with a sublethal dose of PR8 (50 TCID_50_), and 5 days later infected with a sublethal dose of T4 (10^6^ CFU/mouse). Heart rate (**A**), breath rate (**B**), arterial oxygen saturation (SpO_2_, **C**), body weight loss (**D**), and survival rate (E, >30% body weight loss as an endpoint) were measured and recorded on different days after PR8 (PR8, *n* = 12), T4 single infection (T4, *n* = 13), or PR8 and T4 coinfection (PR8/T4, *n* = 12). Bacterial (**F**) and viral (**G**) loads in lung homogenate on the indicated days after PR8/T4 infection. dpi=day post-infection. Data are mean ± s.e.m. from 5 to 13 mice in each group. ****P < 0.0001; ***P < 0.001; ^NS^*P* > 0.05.

Before establishing suitable coinfection models, we characterized a sublethal PR8 (H1N1) IAV single infection which induces minimal clinical signs of flu. Following intranasal infection, PR8-infected mice started losing body weight by days 4 and 5 postinfection. At 50, 100, and 200 times the 50% tissue culture infective dose (TCID_50_) PR8 infection induced maximum weight loss on day 8 of about 11%, 17%, and 26%, respectively (Fig. S1). By day 15, all mice recovered 100% of their original body weights. Decrease in blood oxygen saturation is associated with major adverse events in patients with pneumonia (42), and blood oxygen levels are more accurate than body weight loss in predicting lung pathology in hosts infected with influenza virus (43). Arterial oxygen saturation has been used to assess lung function in mice infected with IAV (44) or *Sp* (45). After 50 TCID_50_ PR8 infection, the heart rate, breath rate, and arterial O_2_ saturation of individual mice were measured using noninvasive, real-time pulse oximetry (MouseOx, STARR Life Sciences, Oakmont) (Fig. 1A–C). At the peak of weight loss, mice remained at about 90% O_2_ saturation on day 8 after PR8 infection. Heart and breath rates were slightly decreased in mice given the lowest dose of PR8 (50 TCID_50_). These results show that this low dose of IAV does not induce severe lung dysfunction and respiratory distress in mice, which may more accurately reflect low-dose flu infection in humans compared to the higher doses of IAV typically used in experimental infections.

To determine the effects of prior low-dose flu infection on secondary *Sp* infection, mice were intranasally infected with low-dose PR8 (50 TICD_50_) and 5 days later challenged with a sublethal dose (10^6^ CFU) of *Sp* TIGR4 (T4) of serotype 4 in comparison to control mice receiving the same doses of PR8 or T4 alone. Mice remained at >95% O_2_ saturation along with slightly decreased heart and breath rate during T4 single infection (Fig. 1A–C). T4 singly infected-mice lost only ~6% body weight at days 1 or 2 after infection and then recovered to their original body weights by day 6 (Fig. 1D). Conversely, PR8 and T4 coinfection significantly increased animal morbidity and mortality (Fig. 1). Particularly, O_2_ saturation levels declined to ~70% at 8/3 days after PR8/T4 coinfection (Fig. 1C), along with significantly decreased heart and breath rates (Fig. 1A and B). At 11/6 days after coinfection, body weights of surviving animals were less than 70% of their original body weights (Fig. 1D). Note that mice infected with PR8 or T4 alone showed transient weight loss and displayed minimal signs of clinical disease, and no animal succumbed to single infection. Conversely, coinfection with PR8/T4 induced severe lung dysfunction, respiratory distress, and 100% mortality in mice (Fig. 1E).

To further assess the host defense against secondary pneumococcal infection, pulmonary bacterial burdens were determined 1 and 2 days after *Sp* infection. Consistent with the severity of disease, bacterial loads were significantly higher in coinfected mice compared with *Sp* singly infected mice (Fig. 1F). On day 2 after *Sp* infection, bacteria were undetectable in lungs of mice with *Sp* single infection but remained abundant in the lungs of mice with coinfection, suggesting that bacterial clearance is impaired during coinfection. Viral load was determined by real-time quantitative PCR in lung homogenates 7 days after PR8 infection. Viral loads were comparable in the lungs of mice with PR8 monoinfection and PR8/T4 coinfection (Fig. 1G), indicating that antiviral immunity was not affected during coinfection. Taken together, these results of increased morbidity and mortality, bacterial outgrowth, and comparable virus load in coinfection clearly show that even a low-dose viral infection increases susceptibility to secondary bacterial infection.

### *Sp*-reactive Th17 responses were inhibited during coinfection

**Fig 2:**
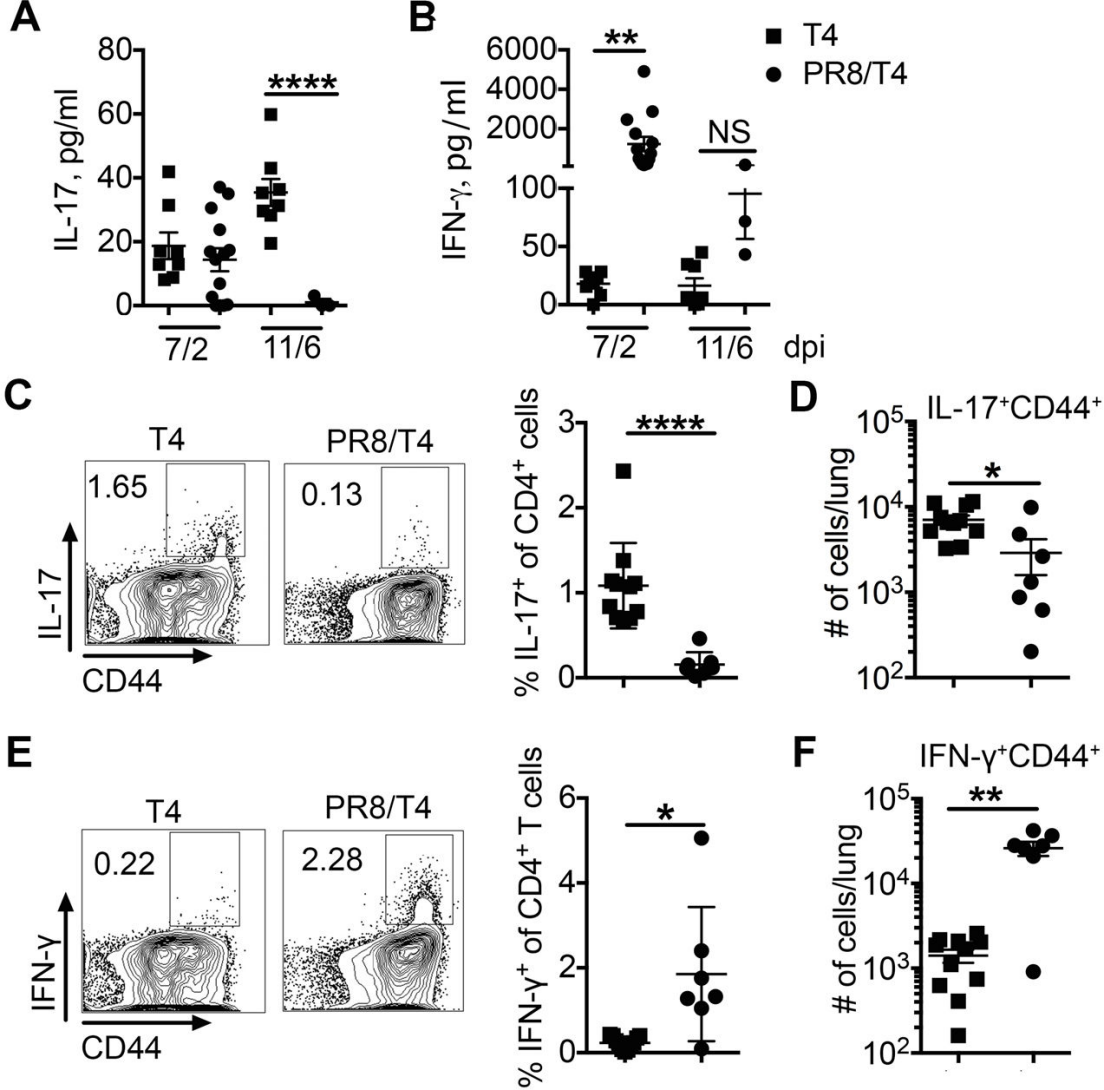
Bacteria-specific T-helper type 17 (Th17) responses are inhibited in the lung during virus and bacterial coinfection. Cytokines interleukin-17A (IL-17A, **A**) and interferon-γ (IFN-γ, **B**) in bronchoalveolar lavage fluid (BALF) on days 2 and 6 after T4 infection in T4 and PR8/T4 infected mice. IL-17A (**C, D**) and IFN-γ (**E, F**) production by CD4^+^T cells after stimulation with heat-killed T4 as (**C, E**) visualized by FACS and (**D, F**) calculated as the number of IL-17A^+^CD44^+^ and IFN-γ^+^CD44^+^ producing CD4^+^T cells per lung on 6 days after T4 infection. Data are mean ± s.e.m. from 3 to 13 mice in each group. ****P < 0.0001; **P < 0.01; *P < 0.05; ^NS^*P* > 0.05. FACS, fluorescence-activated cell sorting.

Type 17 immunity plays an important role in controlling infection by extracellular bacterial pathogens, including *Sp,* through IL-17A production (31). To investigate whether prior influenza infection diminished host antibacterial defense via affecting the Type 17 immune response in the lung, IL-17A and IFN-γ were determined in BALF from mice infected with T4 and PR8/T4 at days 7/2 and 11/6. IL-17A cytokine levels were not significantly different between the two groups at an early time (2 days) after T4 infection, while more IL-17A cytokine was induced in mice at day 6 in T4-infected mice compared to coinfected mice (Fig. 2A), suggesting that the adaptive Th17 cell response at a later time point was inhibited in coinfection. In contrast, there was an increase in IFN-γ in BALF of coinfected mice compared with T4 singly infected mice (Fig. 2B). Taken together, these results indicate that coinfection induces a robust Th1 response and inhibits the Th17 response.

To further evaluate Th1 and Th17 responses in mice infected with T4 alone versus PR8/T4, we analyzed expression of IL-17A^+^CD4^+^ T cells in lung lymphocytes by intracellular cytokine staining following *in vitro* stimulation with PMA/ionomycin 6 days after bacterial infection. There was a high percentage (~2.8%) of IL-17A^+^CD4^+^ T cells from lungs of T4-infected mice, whereas a smaller percentage (1.1%) of them was present in coinfected mice, accompanied with a comparable total number of IL-17A^+^CD4^+^ T cells in coinfected mice (Fig. S2A). In contrast, there was a higher percentage (~10-fold) and total number (~100-fold) of IFN-γ^+^CD4^+^ in lungs of coinfected mice compared with those of T4 singly infected mice (Fig. S2B). To characterize bacteria-reactive T cell responses, we analyzed Th17 and Th1 responses following *in vitro* stimulation with heat-killed T4. There was a markedly reduced number and frequency of T4-reactive IL-17A^+^CD4^+^ T cells in the lungs of coinfected mice compared to T4 singly infected mice (Fig. 2C and D). In contrast, the number of IFN-γ^+^CD4^+^ T cells responding to T4 was significantly higher in mice with coinfection compared to T4 single infection (Fig. 2E and F).

Both Th1 and Th17 responses have been shown to have important roles in controlling lung infection (31, 46). The results above show that the Th17 response is inhibited, while the Th1 response is increased in coinfection compared with bacterial single infection. Of particular importance, preceding influenza infection specifically inhibited the protective *Sp*-reactive Th17 response. Taken together, these data illustrate a potential mechanism by which IAV suppresses the bacteria-specific Th17 response in the lung.

### Prior *Sp* infection protected against viral and bacterial coinfection

**Fig 3:**
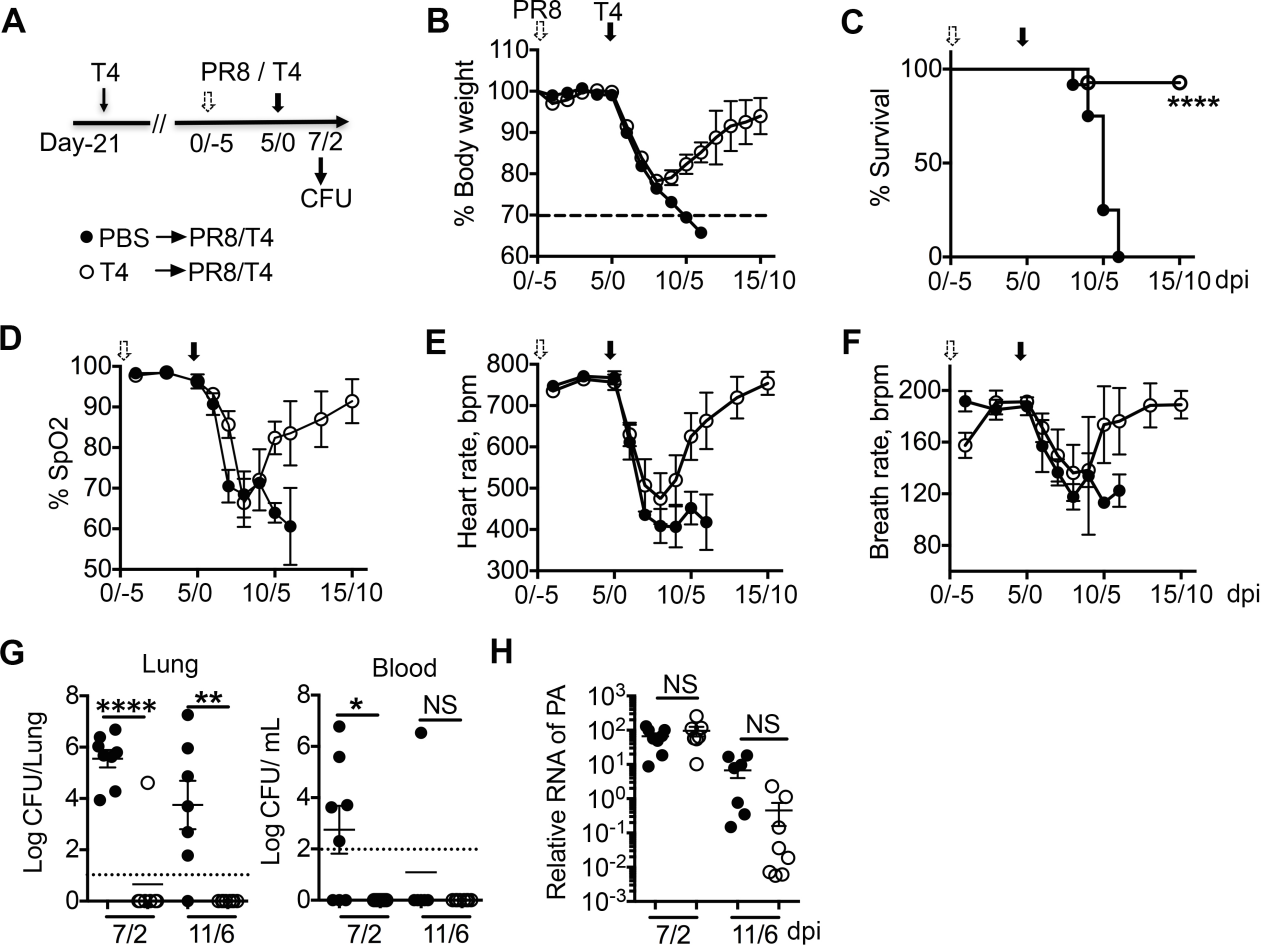
Prior *Sp* infection protects against virus and bacterial coinfection. Mice were inoculated with T4, and 21 days later, were first infected with PR8 and secondarily infected with T4 (**A**). Body weight loss (**B**), survival rates (**C**, >30% body weight loss as an endpoint), arterial oxygen saturation (SpO_2_, **D**), heart rate (**E**), and breath rate (**F**) on different days after PR8/T4 infection in PBS (*n* = 12) or T4 preinfected mice (*n* = 14). Bacterial (G, in lung and blood) and viral loads (**H**) in lung homogenates of PBS or T4 preinfected mice on the indicated days after PR8/T4 infection. Data are mean ± s.e.m. from 5 to 10 mice in each group. ****P < 0.0001; **P < 0.01; *P < 0.05; ^NS^*P* > 0.05. PBS, phosphate-buffered saline.

Previous studies by our laboratory and others have shown that intranasal immunization with bacteria induces memory Th17 responses and provides protection against bacterial reinfection (32, 33). Considering the inhibition of bacteria-reactive Th17 responses in coinfection and the high plasticity of Th17 cells (37), we next examined the protective efficacy of memory Th17 cells elicited by *Sp* preinfection against secondary *Sp* infection in the context of the Th1 inducing environment occurring during IAV infection, and whether *Sp* preinfection can induce bacteria-specific Th17 responses that endure in coinfection. Mice were intranasally inoculated with 10^6^ CFU live T4 or PBS as a mock control and then 21 days later infected with a sublethal dose (50 TCID_50_) of PR8 followed by superinfection with T4 (10^6^ CFU) 5 days after virus infection (Fig. 3A). Mice that had resolved a prior T4 infection and control mice lost body weight at a similar rate during the first 3 days after coinfection. T4 preinfected mice regained body weight 4 days after coinfection and fully recovered to their original body weight by approximately 10 days after coinfection. In contrast, control mice continuously lost their body weight and completely succumbed to coinfection (Fig. 3B). As expected, 100% of PBS mice succumbed to coinfection in 3–6 days but most (90%) of the preinfected mice survived (Fig. 3C). Consistent with body weight loss, arterial O_2_ saturation (Fig. 3D), heart rate (Fig. 3E), and breath rate (Fig. 3F) were decreased in the first 3 days after coinfection in both PBS and T4 preinfected mice. However, T4 preinfected mice started to recover O_2_ saturation, heart and breath rates on day 4 and were almost fully recovered by day 10 after coinfection. These results indicate that lung function rapidly recovers in T4 preinfected mice. Thus, prior infection with *Sp* significantly decreases morbidity and mortality caused by coinfection with virus and *Sp*.

We next determined whether the protection induced by prior bacterial infection was due to rapid clearance of bacteria. Bacterial loads in the lung and blood were determined at 7/2 and 11/6 days after PR8/T4 coinfection. In contrast to outgrowth of bacteria in the lungs of nonimmune mice (~10^5^ CFU), almost all T4 preinfected mice had cleared bacteria from their lungs by day 7/2 after PR8/T4 coinfection (Fig. 3G). By day 11/6 after PR8/T4 coinfection, there were no detectable bacteria in the lungs of any of the T4 preinfected mice; however, most of the PBS mice still had bacteria in their lungs (~10^4^ CFU). In contrast to T4 preinfected mice, which had no detectable bacteria in the blood, most (62.5%) of the PBS mice with coinfection had bacteria in the blood by day 7/2 after PR8/T4 infection (Fig. 3G). Surviving PBS mice cleared bacteria in the blood by 11/6 days after PR8/T4 coinfection. These results indicated that prior T4 infection completely protects mice from bacteremia. We also measured viral loads on 7/2 and 11/6 days after coinfection to determine the effect of T4 immunization on viral clearance. Viral loads in lungs of PBS and T4 preinfected mice were similar on day 7/2 and on day 11/6 after coinfection there was a slight decrease that was not statistically significant (Fig. 3H). Therefore, prior T4 infection protected mice from PR8/T4 coinfection mainly by clearance of bacteria, but also potentially contributed to viral clearance.

### Prior *Sp* infection increased *Sp*-reactive Th17 recall responses in viral and bacterial coinfection

**Fig 4:**
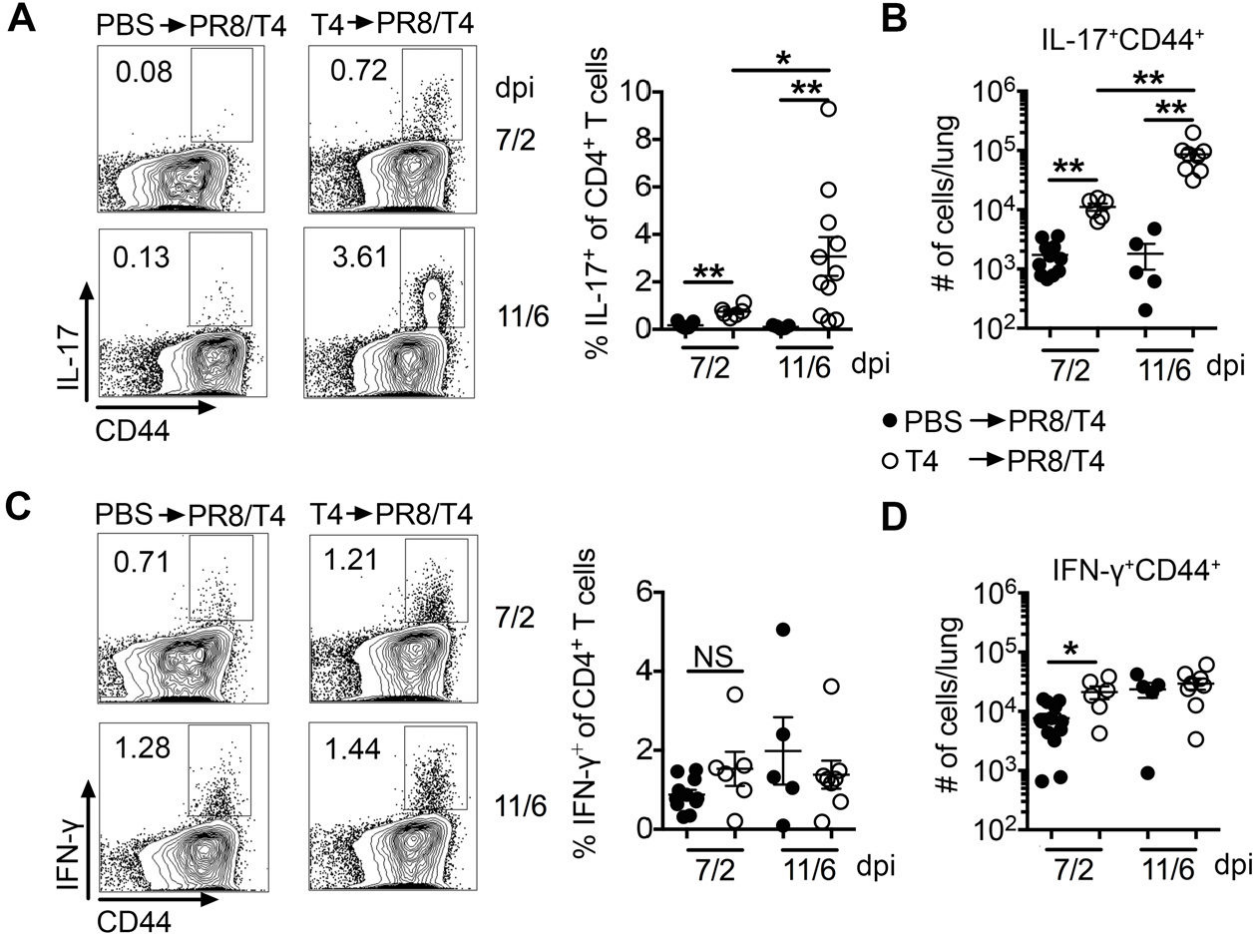
Prior *Sp* infection increases *Sp*-specific Th17 responses in virus and bacterial coinfection. IL-17A (**A, B**) and IFN-γ (**C, D**) production by CD4^+^T cells after stimulation with heat-killed T4 as (**A, C**) visualized by FACS and (**B, D**) calculated as the number of IL-17A^+^CD44^+^ and IFN-γ^+^CD44^+^ producing CD4^+^T cells per lung on the indicated days of PR8/T4 infection in PBS or T4 preinfected mice. Data are mean ± s.e.m. from 5 to 11 mice in each group. **P < 0.01; *P < 0.05; ^NS^*P* > 0.05. FACS, fluorescence-activated cell sorting; PBS, phosphate-buffered saline.

To determine whether prior *Sp* infection could induce *Sp* reactive Th17 recall responses in mice during coinfection, we analyzed IL-17A expressing CD4^+^ T cells by intracellular cytokine staining following *in vitro* stimulation with heat-killed T4 of T-cells isolated from mice 7/2 and 11/6 days after coinfection. By 7/2 and 11/6 days after coinfection, there was a higher percentage and total number of CD4^+^ T cells expressing IL-17A from lungs of T4 preinfected mice compared to that of nonimmune mice (Fig. 4A and B). Interestingly, there was also a significant increase in the percentage (~5-fold) and total number (~10-fold) of IL-17A^+^CD4^+^ T cells on day 11/6 compared to day 7/2 after coinfection in the lungs of T4 preinfected mice. In sharp contrast, the percentage and total number of IL-17A^+^CD4^+^ T cells were comparable between days 7/2 and 11/6 after coinfection in unimmunized mice. These data indicate that prior *Sp* infection induced memory Th17 cell expansion during coinfection. The percentage and total number of IFN-γ^+^CD4^+^ T cells in lungs of T4 preinfected mice were similar to those of unimmunized mice on day 11/6 of coinfection, with a small but significant difference in absolute numbers only on day 2 (Fig. 4C and D). These results indicate that prior *Sp* infection has a minor effect on bacteria-primed Th1 responses compared with Th17 responses to *Sp* in the lungs of coinfected animals.

We also analyzed total Th1 and Th17 cells in the lungs of unimmunized and T4 preinfected mice on days 7/2 and 11/6 of coinfection by intracellular cytokine staining following *in vitro* stimulation with PMA/ionomycin. Percentages and numbers of IL-17A-producing CD4^+^ T-cells significantly increased in T4 preinfected mice compared with PBS control mice on days 7/2 and 11/6 of coinfection (Fig. S3A). Percentage and numbers of IFN-γ-producing CD4^+^ T were similar in the lungs of T4 preinfected and PBS mice on days 7/2 and 11/6 of coinfection (Fig. S3B). These results are consistent with the data above, which showed that prior *Sp* infection primarily enhanced the bacteria-primed Th17 response with only a minor effect on the Th1 response.

### Protection from secondary bacterial infection induced by prior *Sp* infection is dependent on IL-17A

**Fig 5:**
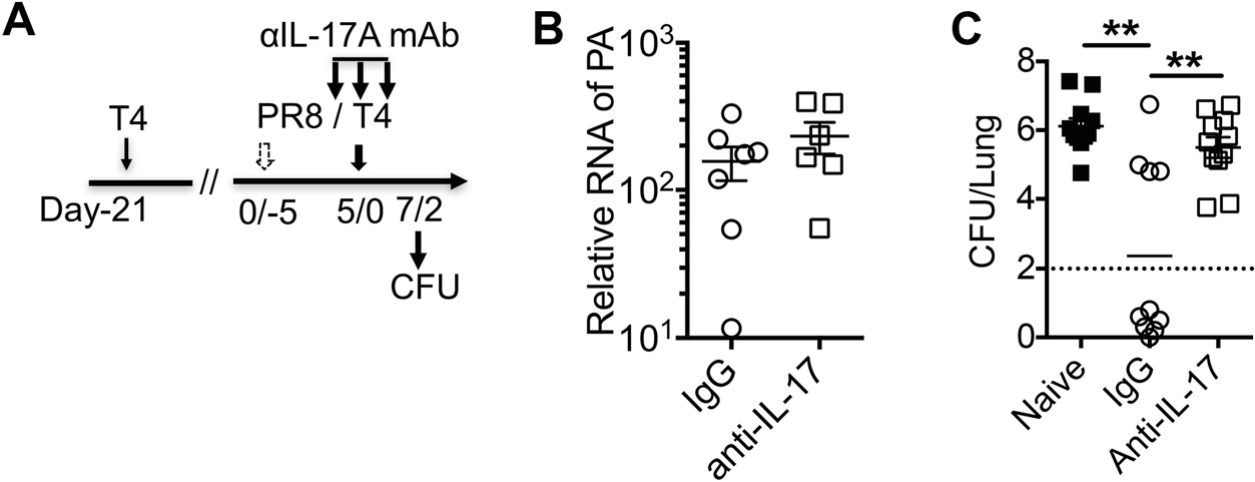
Protection from coinfection induced by prior *Sp* infection is dependent on IL-17A. At 21 days postinoculation with T4, B6 mice were first infected with PR8 and secondarily infected with T4 (**A**). Preinfected mice also treated with IL-17-neutralizing antibody (anti-IL-17, clone 17F3) or isotype control antibody (IgG) intraperitoneally on days −1, 0, and 1 and intranasally on day 0 during T4 challenge following PR8 infection. Virus loads (**B**) and bacterial loads (**C**) in lung homogenate of naïve or T4 preinfected mice treated with IL-17-neutralizing antibody (anti-IL-17) or isotype control antibody on 7/2 days after PR8/T4 infection. Data are mean±s.e.m. from 7 to 14 mice in each group. ****P < 0.0001; **P < 0.01; *P < 0.05; ^NS^*P* > 0.05.

Our results have thus far shown that *Sp*-specific Th17 responses in mice during coinfection were rescued by prior infection with *Sp*, correlating with protection. We next further investigated the role of IL-17A in protection against secondary bacterial infection following IAV infection by *in vivo* blockade of IL-17A. Depletion of IL-17A in the BALF of mice was confirmed by ELISA 2 days after coinfection (Fig. S4). T4 preinfected mice were treated with anti-IL-17A antibodies or the isotype control antibody on days −1, 0, and 1 by intraperitoneal injection and intranasally on day 0 during T4 challenge following PR8 infection (Fig. 5A). Viral loads in the lungs of these mice were not significantly affected by IL-17A blockage (Fig. 5B). However, IL-17A neutralization led to a significant increase in bacterial loads in the lungs compared to that with isotype control antibody treatment (Fig. 5C). Thus, blockade of IL-17A abrogated the protective effect of prior T4 infection, which indicates that protection against secondary bacterial infection induced by prior *Sp* infection is dependent on IL-17A.

### Prior *Sp* infection induces a cross-reactive Th17 response and provides heterologous protection against coinfection

We and others have demonstrated that the memory Th17 response is a major predisposing factor in immunization-derived protection against single bacterial infection with heterologous strains (32, 33). Therefore, we further investigated whether prior bacterial infection induces Th17-mediated cross-protection against coinfection. Mice were inoculated with 10^6^ CFU of a low virulence *Sp* strain, P1121 (serotype 23F) (47, 48). We analyzed IL-17A expressing CD4^+^ T cells in the lung at 6 and 21 days after infection by intracellular cytokine staining following *in vitro* stimulation with heat-killed P1121, T4 and a gram-negative bacterium, *H. influenzae* strain NT127. On day 6 after P1121 exposure, Th17 cells were capable of responding to both homologous and heterologous heat-killed P1121 and T4, but not *H. influenzae* NT127 (Fig. S5A). These results suggest that *Sp* infection induces robust cross-reactive Th17 responses to different serotypes of *Sp*. On day 21 after P1121 infection, there were antigen-specific memory Th17 cells responding to both P1121 and T4. We further tested memory Th17 responses in the lung at a later time (day 56) post P1121 infection following stimulation with heat-killed P1121 and characterized the phenotype of memory Th17 cells on days 21 and 56. Most of the P1121-specific Th17 cells on both days had differentiated into the tissue-resident memory (T_RM_)-expressing CD69^hi^/CD62L^lo^ phenotype (Fig. S5B). In contrast to Th17 responses, the IgG antibody response in P1121-infected mice reacted only with P1121 but not with heterologous T4 or other bacteria, NT127 (Fig. S5C). To further test if the protection against IAV/*Sp* coinfection requires memory Th17 cells specific to *Sp*, we infected mice with NT127 and 21 days later challenged the mice with PR8/T4 coinfection (Fig. S6A). Memory Th17 responses to NT127 in the lung on day 21 post NT127 infection were examined by intracellular cytokine staining following heat-killed NT127 stimulation. Although there were memory Th17 cells reactive to NT127 (Fig. S6B), mice previously infected with NT127 had similar bacterial burdens in the lung compared with PBS mice (Fig. S6C). Taken together, these results demonstrate that *Sp* preinfection induces cross-reactive Th17 cells against different serotypes of *Sp* but not antibodies, which indicates that *Sp* preinfection may provide cross-serotype protection against coinfection by a Th17-dependent mechanism.

**Fig 6:**
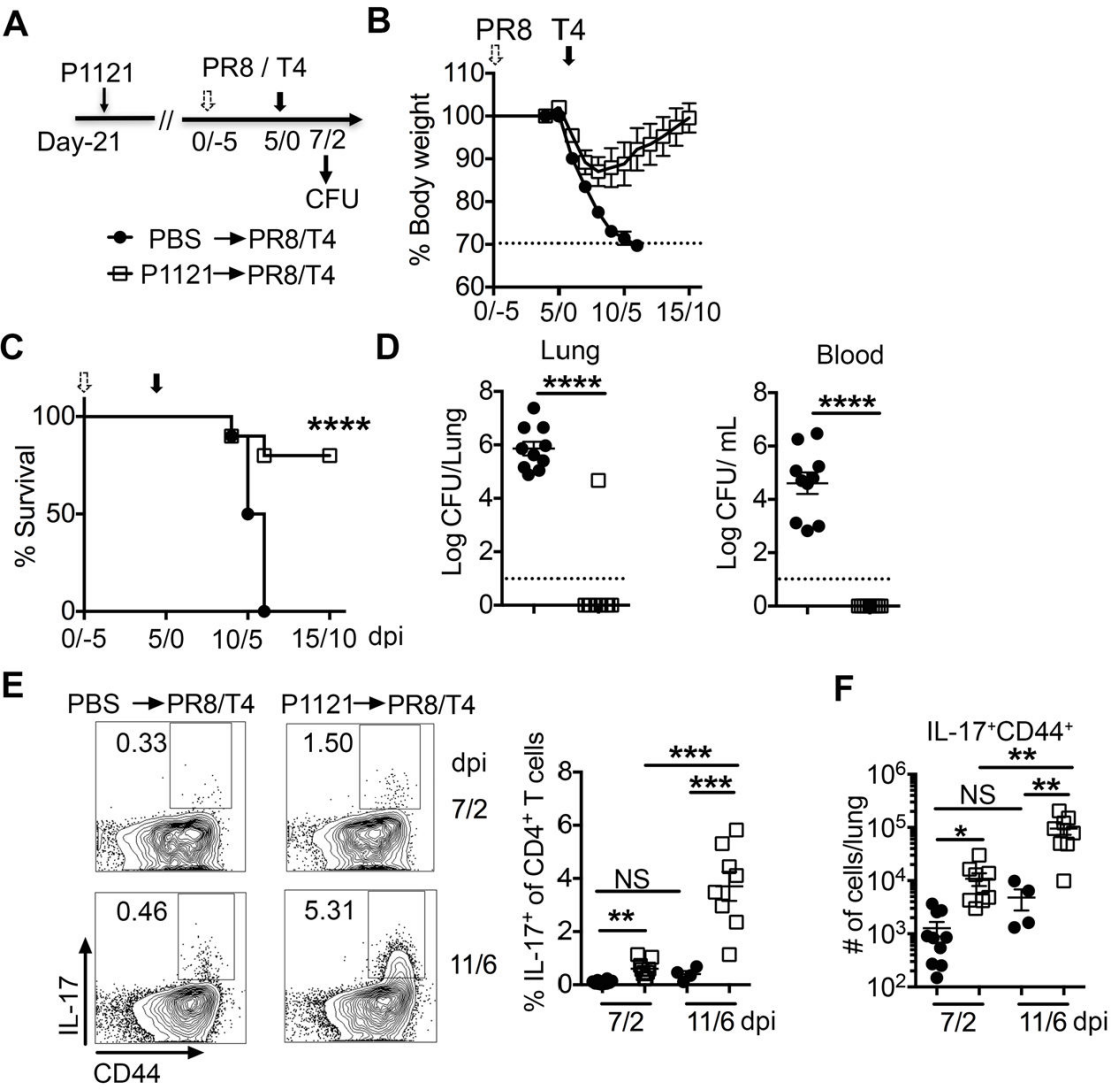
Prior *Sp* infection induces a cross-specific Th17 response and provides heterologous protection against coinfection. At 21 days postexposure with P1121, mice were first infected with PR8 and secondarily infected with T4 (**A**). Body weight loss (**B**) and survival rates (C, >30% body weight loss as an endpoint) on the indicated days after PR8/T4 infection in PBS (*n* = 10) or T4 preinfected mice (*n* = 10). Bacterial loads (**D**) in lung homogenate and blood of PBS or T4 preinfected mice on 7/2 days after PR8/T4 infection. IL-17A production by CD4^+^ T cells after stimulation with heat-killed T4 as visualized by FACS (**E**) and (**F**) calculated as the number of IL-17A^+^CD44^+^ and IFN-γ^+^CD44^+^ producing CD4^+^ T cells per lung on the indicated days after PR8/T4 infection in PBS or T4 preinfected mice. Data are mean±s.e.m. from 4 to 10 mice in each group. ****P < 0.0001; ***P < 0.001; **P < 0.01; *P < 0.05; ^NS^*P* > 0.05. FACS, fluorescence-activated cell sorting; PBS, phosphate-buffered saline.

To further evaluate prior *Sp* infection induced cross-protection against coinfection, mice were intranasally inoculated with 10^6^ CFU live P1121 or PBS for control, and 21 days later infected with a sublethal dose of PR8 followed by superinfection with T4 (Fig. 6A). Mice previously infected with P1121 and control mice lost body weight at a similar rate during the first 3 days of coinfection. However, P1121 preinfected mice regained body weight by 4 days of coinfection and fully recovered to their original body weight approximately 10 days after coinfection. In contrast, control mice continuously lost body weight and succumbed to coinfection (Fig. 6B). P1121 preinfection significantly increased the survival rate of mice during coinfection compared to control mice (Fig. 6C). On day 7/2 of coinfection, bacteria were undetectable in the lungs and blood of P1121 preinfected mice but were abundant in these sites of control mice (Fig. 6D). Similar to homologous T4 preinfection, virus loads were comparable in P1121 preinfected and PBS mice (data not shown). On days 7/2 and 11/6 of coinfection, there was a dramatically higher percentage and total number of Th17 cells responding to T4 (Fig. 6E and F) and P1121 (data not shown) from lungs of P1121 preinfected mice compared with that of control mice. The percentage and total number of IL-17A^+^CD4^+^ T cells were significantly increased on day 11/6 compared to day 7/2 of coinfection in the lungs of P1121 immunized mice. Taken together, these data suggest that the Th17 recall response by memory CD4^+^T cells may have an important role in cross-protection against coinfection with different serotypes of bacteria following IAV infection.

## Discussion

Bacterial coinfection with IAV remains a significant cause of hospitalizations and mortality worldwide (49 - 51). The underlying immune mechanisms of IAV/bacterial coinfection are not fully understood, especially the aspects of adaptive immune responses which are crucial for vaccine development. Since most influenza patients have asymptomatic/mild and self-limited illnesses, we refined a coinfection model with sublethal doses of IAV which induced only mild clinical signs of flu, followed by a secondary *Sp* infection to study the immune mechanism of coinfection and to investigate protective immunity against coinfection in this study. Our data demonstrate that even a mild infection with IAV increases susceptibility and mortality to secondary bacterial infection, and similar to high-dose IAV infection, adaptive bacteria-reactive Th17 responses were dramatically impaired in these coinfections. Importantly, prior infection with *Sp* overcame this susceptibility by rescuing the bacteria-reactive Th17 response and protected mice from viral/bacterial coinfection by promoting clearance of bacteria. Depletion of IL-17A in preinfected mice with an IL-17A-neutralizing antibody abrogated *Sp* preinfection induced protection, suggesting that protection against secondary bacterial infection induced by prior *Sp* infection is dependent on IL-17A. Moreover, our data show that while *Sp* preinfection induces cross-reactive Th17 cells, it fails to induce antibodies against a heterologous strain. Our data further show that *Sp* preinfection provides cross-protection against coinfection with different serotypes of *Sp* following IAV infection. These findings support the value of Th17-based vaccines for prevention of coinfection.

IAV infections are associated with a wide range of clinical manifestations, ranging from asymptomatic infection and critical illness to death (52). There is an average of 19.1% and 43.4% of infections that are asymptomatic and subclinical, respectively (53). A flu watch cohort study showed that influenza infected 18% of unvaccinated people each winter and up to 75% of these infections were asymptomatic (54, 55). In this study, we used a sublethal dose of IAV infection to induce a subclinical flu infection and then coinfected with *Sp*. Our results demonstrated that even low-dose flu infection significantly increased susceptibility and mortality to secondary bacterial infection (Fig. 1). Compared with bacterial single infection, bacterial clearance and lung function were significantly impaired during coinfection. These results suggest that people with asymptomatic IAV infection may still be highly susceptible to secondary bacterial infection. Consistent with this hypothesis, it was recently reported that bacterial detection and density of bacteria increased during asymptomatic viral infection in children, and *Sp* was the bacterial species showing the greatest increases (56). Considering that mortality incidence peaks several days after coinfection (Fig. 1), even people with asymptomatic IAV infection should be vigilantly monitored for secondary bacterial infection.

Multiple immune mechanisms for increased susceptibility to bacterial infections following viral infection have been investigated, including defects in innate and adaptive immune responses. In this study, to investigate the underlying adaptive immune mechanism for increased susceptibility to bacterial infection after IAV infection, we evaluated Th1 and Th17 responses in the lung. Our results showed that bacteria-specific CD4^+^ T cells producing IL-17A, but not those producing IFN-γ, were decreased in coinfection compared with bacterial single infection. This observation is consistent with results found in IAV coinfections with *Staphylococcus aureus* or gram-negative bacteria (*Escherichia coli* and *Pseudomonas aeruginosa*) (36, 57). IAV suppresses Th17 immunity and attenuates Th17-induced antimicrobial peptides, which are necessary for bacterial clearance in the lung (57, 58). Increased type I IFN (57, 58), IL-27 (59), and MicroRNA-155 (60), and decreased IL-1β (61) have been shown to be associated with the attenuation of the bacteria-driven Th17 pathway. Aside from IAV attenuation of the *Sp*-driven Th17 pathway, the IFN-γ response (both total and that produced by *Sp*-specific Th1 cells) was found to be increased in the coinfected mice compared to those with *S*p infection alone (Fig. 2 and Fig. S2), which might contribute to decreased antibacterial immune responses and elevated susceptibility to secondary bacterial infection. This potential mechanism has been supported by a study reporting that IFN-γ inhibits initial bacterial clearance from the lung by alveolar macrophages (27, 62). In contrast to the IAV/*Sp* model, bacteria-specific Th1 responses (IFN-γ) were similar between *H. influenzae* single infection and IAV/*H. influenzae* coinfection despite that Th17 responses specific to *H. influenzae* were inhibited as well (63), suggesting the impairment of antibacterial Th17 responses is modulated by different mechanisms in different bacteria coinfected with IAV. γδ T cells are specialized innate T cells that produce IL-17A and have important roles in protection against bacterial infection (64). Function of IL-17A-producing γδ T cells has been shown to be compromised during secondary *Sp* infection after IAV infection (29, 30). Moreover, impairment of IL-17A-producing γδ T cells was associated with increased type I IFN and IL-27 (65, 66). In these studies, the authors detected decreased IL-17 production at early time points (1 day) after bacterial infection, because γδ T cells exhibited an early burst of IL-17A gene expression, which peaked at 8 h and declined by 24 h after bacterial infection. In our study, we found significantly decreased IL-17 production in the lungs of coinfected mice compared with single bacterial infection at 6 days but not at 2 days of bacterial infection (Fig. 2A). Adaptive Th17 cells are the main source of IL-17 at the later stage of bacterial lung infection (67). The delayed inhibition of IL-17 production likely reflects suppression of the overall Th17 response. Taken together, these results show that bacteria-specific Th17 responses are inhibited in coinfection, which is consistent with results found in IAV coinfection with other bacteria.

It is well known that prior exposure to bacteria or bacterial components can induce bacteria-specific immune memory and provide protection against secondary challenge with the same bacterium. However, mechanisms of protection that are effective in pneumonia caused by coinfection are largely unknown. Our previous results showed that prior lung infection with live *Sp* induced the highest level of protection against *Sp* single infection, better than prior nasal colonization or killed *Sp* immunization (32). In this study, our results showed that prior *Sp* infection provided protection against coinfection by promoting clearance of bacteria and accelerating lung function recovery (Fig. 3). These results indicate that bacterial infection induced immunity is sufficient for protection against coinfection, which is consistent with a previous study showing that preinfection with an attenuated pneumococcal mutant was protective against influenza virus and secondary pneumococcal infection and decreased bacterial burden in the lung (68). In contrast, PCV immunization appears to only provide protection against *Sp* infection alone, with less efficacy against coinfection (11, 14, 69). In addition, mucosal or subcutaneous immunization with fusion protein DnaJ-ΔA146Ply of *Sp* induced antibody responses that were only 30% or 50% protective, respectively, against influenza and *Sp* coinfection (70). Our data and these studies together indicate that antibody-based vaccines need to be improved and alternative immunity is involved in the more effective protection conferred by prior bacterial infection. CD4^+^ T cells have been shown to be indispensable to reduce the density of bacteria by immunization of mice with a whole-cell vaccine in a model of influenza induced pneumococcal otitis media (71). IL-17A-producing CD4 Th17 cell-dependent immunity is important for protection against infections with single *Sp* infection (32). Prior colonization or immunization with heat-inactivated or live bacteria has been shown to provide protection against bacterial single infection by inducing a memory Th17 response in the lungs (32). Considering results in these studies and other reports showing suppression of IL-17A immunity in IAV/*Sp* coinfection, we tested whether protection induced by *Sp* preinfection was a consequence of induction of Th17 responses that are resistant to the inhibition that occurs during coinfection. Indeed, our data revealed that *Sp*-specific Th17 responses were retained in coinfection of *Sp* preinfected mice (Fig. 4) and protection against coinfection was dependent on IL-17A (Fig. 5), whereas preinfection with other bacteria (*H. influenzae*) that induced memory Th17 cells conferred no protection from IAV/*Sp* coinfection (Fig. S6). We note that despite major protection from lethality (Fig. 3) and bacterial clearance in the majority of *Sp* preinfected mice, clearance of bacteria was not observed in a subset of mice at day 7/2 of coinfection (Fig. 5C). Because 90% of preinfected mice eventually recover from coinfection (Fig. 3), it is likely that some mice clear bacteria at time points later than day 2. Unlike our results with *Sp* coinfection, CD4 Th1 memory cells have been shown to be protective against secondary *S. aureus,* another gram-positive bacterium, in the context of influenza virus infection (72), suggesting different immune responses are required to provide protection against different bacteria following influenza virus infection. Interestingly, our concurrent studies with IAV/*H. influenzae* coinfection have demonstrated that prior immunization or infection with *H. influenzae* generates a durable protective Th17 response against this gram-negative bacterium (63). Therefore, predicting whether a Th17 response elicited by prior infection will be protective cannot be generalized based on classification of the target pathogen as gram-positive versus gram-negative. It is possible that *Sp* and *H. influenzae* require similar host responses for clearance because they inhabit a more similar niche compared with that of *S. aureus*. We further detected cross-protection conferred by prior *Sp* exposure against coinfection with *Sp* of a different serotype. Our results showed that prior infection with the less virulent strain P1121 induced Th17 cells, but not antibodies, that cross-reacted with *Sp* of a different serotype, strain T4, and provided cross-protection against coinfection with this strain (Fig. 6 and Fig. S3). It has been shown that bacterial immunization and colonization induce Th17 cell-dependent, and antibody-independent, cross-serotype protection in single-strain heterologous challenge with bacteria in *K. pneumoniae* and pneumococcal models (33, 73). Previous studies in a model of *K. pneumoniae* infection have shown that, although γδ-T cells were the major source of IL-17 during primary infection, CD4^+^ T cells were primarily responsible for the increased IL-17^+^ cell population observed following immunization (33). Our previous study further revealed the direct role of memory Th17 cells in cross-protection against pneumonia by adoptively transferring memory Th17 from immunized mice to naïve mice (32). Taken together, our data indicate a key mechanism by which the induction and maintenance of bacteria-specific Th17 responses provides broad protection against pneumococcal coinfection and could provide a potential therapeutic strategy for coinfection.

Although our study has shown that memory Th17 cells can overcome IAV-mediated impairment of antibacterial responses and provide protection against subsequent coinfection, the mechanisms of memory Th17-mediated protection in this setting remain unclear. Studies have shown that alveolar macrophages are inhibited during bacterial clearance in the context of influenza infection (27, 74). In addition, the chemotaxis of neutrophils and macrophages is impaired during influenza infection, which contributes to impairment of bacterial clearance in the lung (62, 75, 76). Therefore, the ability of memory Th17 responses to overcome IAV-mediated inhibition of antibacterial immunity may involve rescuing impaired functions of macrophages and neutrophils. The precise underlying mechanisms will require further investigation.

In conclusion, low-dose IAV infection suppressed bacteria-specific Th17 cells and increased susceptibility to secondary bacterial infection. Prior bacterial infection induced Th17-dependent protection against coinfection. Cross-protection conferred by this response indicates that Th17-based vaccines are likely to prove more versatile than traditional antibody-based vaccines. Furthermore, since *Sp* and *H. influenzae* are common agents of secondary bacterial pneumonia in flu-infected patients (77) and a memory Th17 response was effective for both, a combined vaccine can be envisioned that would incorporate an adjuvant to drive a Th17 response together with *Sp* and *H. influenzae* antigens.

## Data Availability

All data are included in the manuscript and/or supporting information.

## SUPPLEMENTAL MATERIAL

Fig. S1 mbio.00519-23-s0001.tif Low-dose flu infection. Mice were infected with different sublethal doses of PR8 (50, 100 and 200 TCID50). Body weight loss was measured on different days after PR8 infection. Data are mean ± s.e.m. from 7-11 mice in each group.

Fig. S2 mbio.00519-23-s0002.tif Th17 and Th1 response in lung during virus and bacterial coinfection. IL-17A (A) and IFN-γ (B) production by CD4+T cells after stimulation with PMA/IONO as visualized by FACS and calculated as the number of IL-17A+CD44+ (A) and IFN-γ+CD44+ (B) producing CD4+T cells per lung on day 6 after T4 infection. Data are mean±s.e.m. from 7-10 mice in each group. ****P* < 0.001; ***P* < 0.01.

Fig. S3 mbio.00519-23-s0003.tif Sp preinfection increases Th17 responses in virus and bacterial coinfection. IL-17A (A) and IFN-γ (B) production by CD4+T cells after stimulation with PMA/IONO as visualized by FACS and calculated as the number of IL-17A+CD44+ (A) and IFN-γ+CD44+ (B) producing CD4+T cells per lung on different days after PR8/T4 infection in PBS and T4 preinfected mice. Data are mean ± s.e.m. from 5-11 mice in each group. PBS and T4 preinfected groups without asterisks are all not statistically different (NS). *****P* < 0.0001; **P* < 0.05.

Fig. S4 mbio.00519-23-s0004.tif IL-17 neutralization. T4-preinfected mice were first infected with PR8 and secondarily infected with T4. Preinfected mice were treated with anti-IL-17A antibody (anti-IL-17, clone 17F3) (*n* = 11) or the isotype control antibody (*n* = 10) intraperitoneally on days -1, 0 and 1 and intranasally on day 0 in comparison to naïve mice (*n* = 14) during T4 challenge following PR8 infection. The level of IL-17A in the BALF of mice was confirmed by ELISA 2 days after coinfection. Data are mean ± s.e.m.. **P* < 0.05.

Fig. S5 mbio.00519-23-s0005.tif Sp preinfection induces cross-reactive memory Th17 cells against different Sp serotypes. Percentage of IL-17A producing CD4+T cells after stimulation with heat-killed Sp (P1121 strain) and Sp (T4 strain) or H. influenzae (NT127 strain) on different days after P1121 exposure in P1121 infected mice or naïve mice (A). The phenotype of P1121-specific memory Th17 cells were examined in the lung on different days after P1121 infection according to their CD62L vs. CD69 expression (B). BALF immunoglobulin G (IgG) against different Sp (P1121 and T4) or NT127 from P1121 preinfected mice (C). Data are mean±s.e.m. from 5 mice in each group. *****P* < 0.0001; ****P* < 0.001; ***P* < 0.01.

Fig. S6 mbio.00519-23-s0006.tif Prior infection with *H. influenzae* does not provide protection against subsequent IAV/Sp coinfection. Mice were previously infected with *H. influenzae* (NT127 strain), and 21 days later challenged with PR8 followed by by Sp (T4 strain) infection (A). On day 21 post NT127 infection, IL-17 production by CD4+ T cells in the lung after stimulation with heat-killed NT127 was visualized by FACS (B). Bacterial loads in the lung on day 7/2 post PR8/T4 coinfection (C). Data are mean±s.e.m. from 4 mice in each group. NSP>0.05.

ASM does not own the copyrights to Supplemental Material that may be linked to, or accessed through, an article. The authors have granted ASM a non-exclusive, world-wide license to publish the Supplemental Material files. Please contact the corresponding author directly for reuse.
