# Supplementary figures and images for: Memory Th17 cell-mediated protection against lethal secondary pneumococcal pneumonia following influenza infection
Source: mBio. 2023 May 24;14(4):e00519-23. doi: 10.1128/mbio.00519-23 (PMC10470593; doi:10.1128/mbio.00519-23)

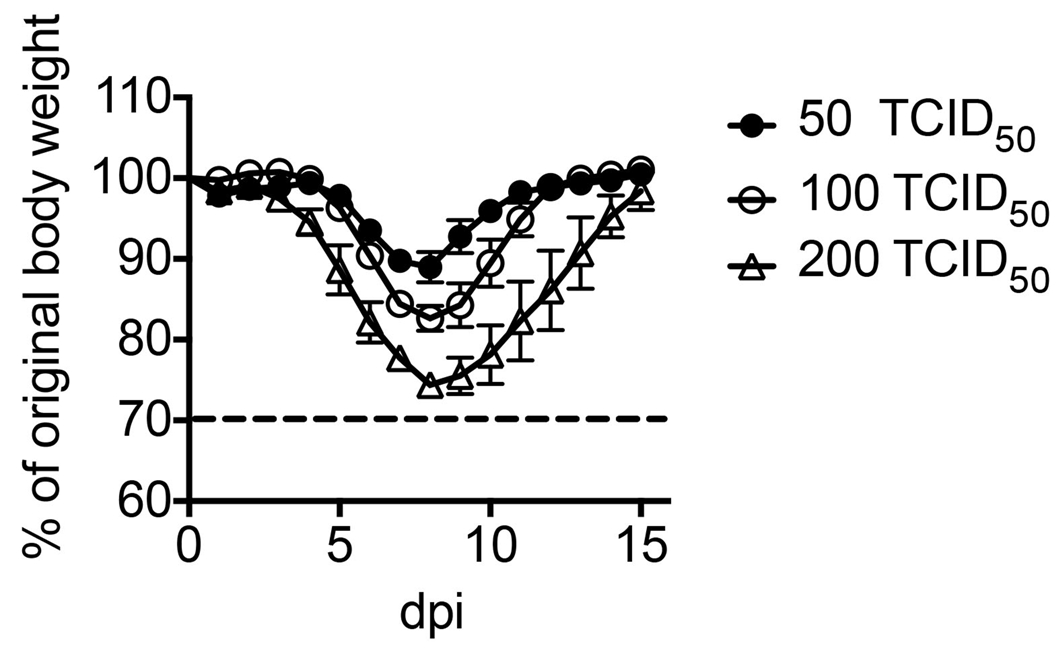

Supplement: Fig. S1 — Low-dose flu infection. Mice were infected with different sublethal doses of PR8 (50, 100 and 200 TCID50). Body weight loss was measured on different days after PR8 infection. Data are mean ± s.e.m. from 7-11 mice in each group. [file mbio.00519-23-s0001.tif]

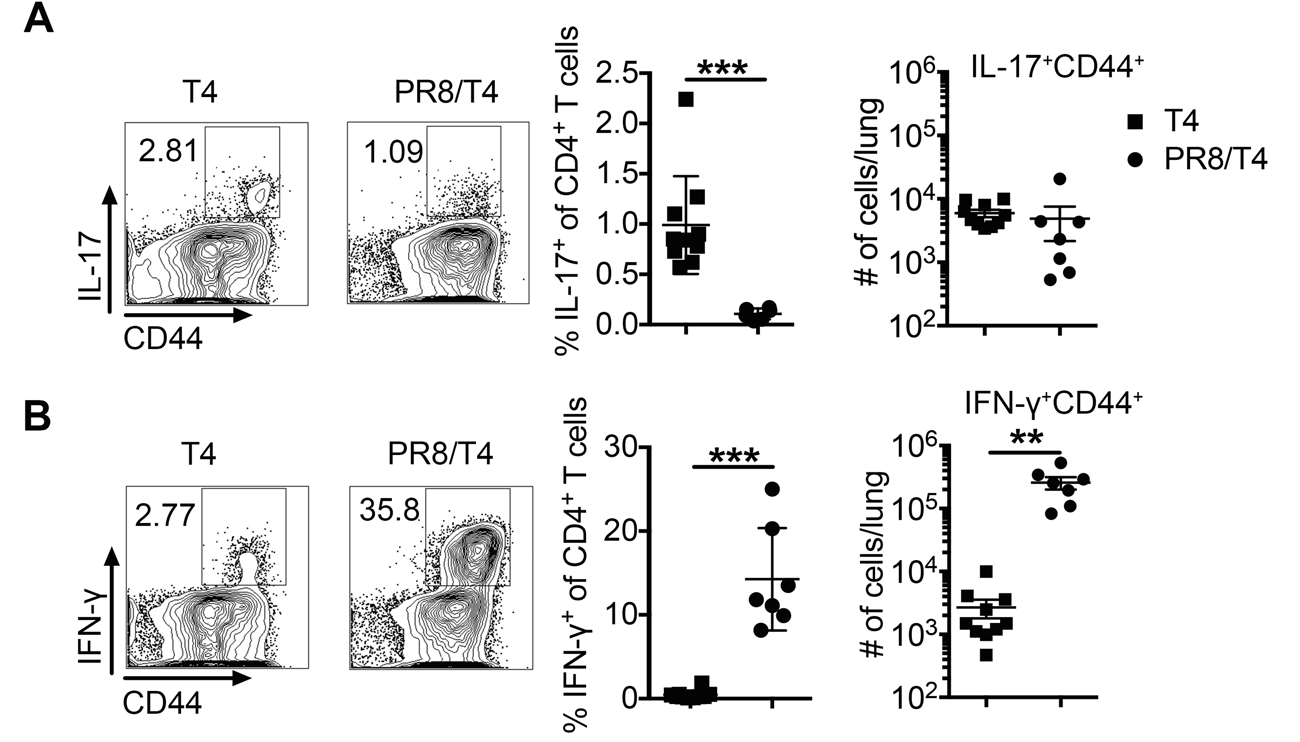

Supplement: Fig. S2 — Th17 and Th1 response in lung during virus and bacterial coinfection. IL-17A (A) and IFN-γ (B) production by CD4+T cells after stimulation with PMA/IONO as visualized by FACS and calculated as the number of IL-17A+CD44+ (A) and IFN-γ+CD44+ (B) producing CD4+T cells per lung on day 6 after T4 infection. Data are mean±s.e.m. from 7-10 mice in each group. ***P < 0.001; **P < 0.01. [file mbio.00519-23-s0002.tif]

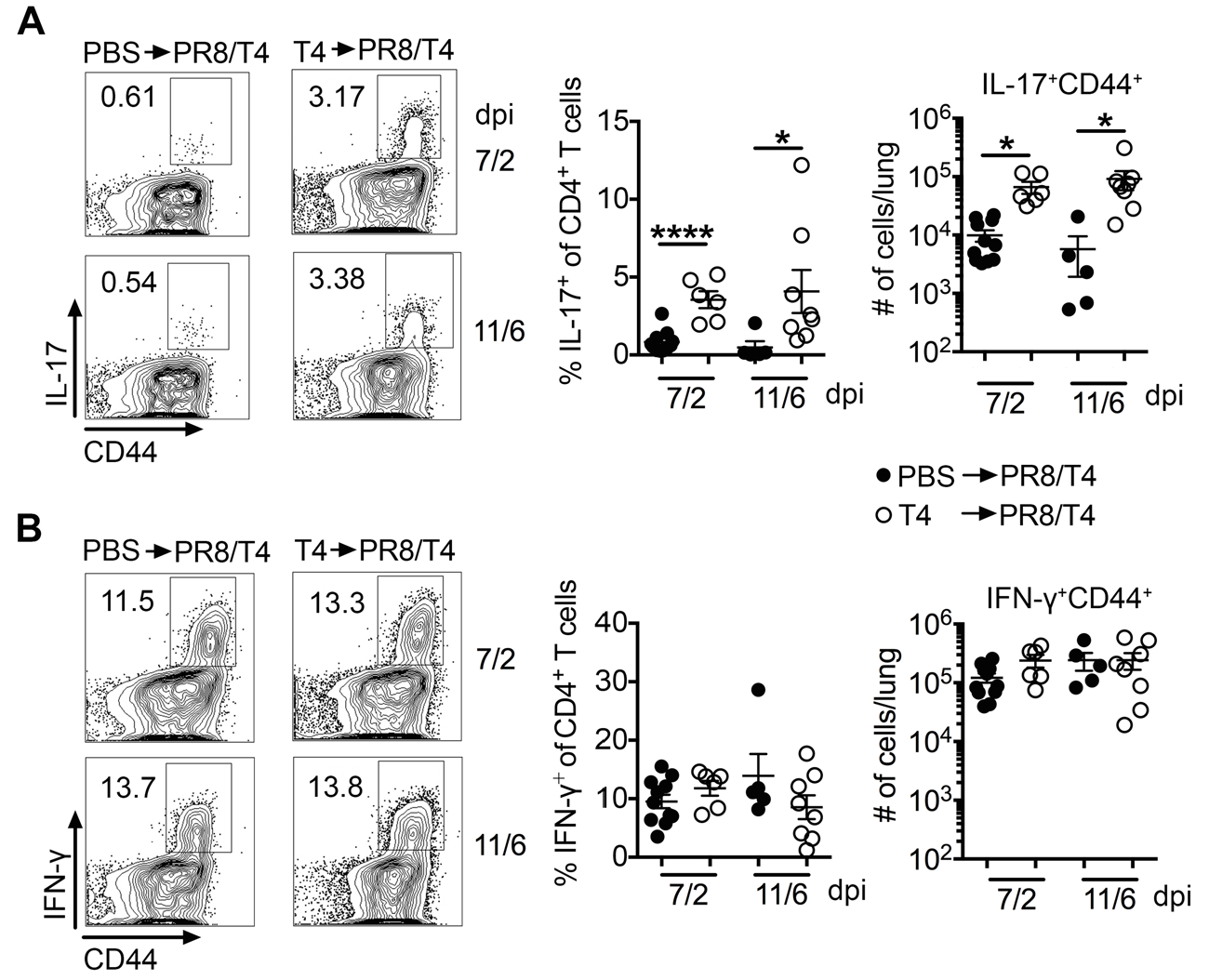

Supplement: Fig. S3 — Sp preinfection increases Th17 responses in virus and bacterial coinfection. IL-17A (A) and IFN-γ (B) production by CD4+T cells after stimulation with PMA/IONO as visualized by FACS and calculated as the number of IL-17A+CD44+ (A) and IFN-γ+CD44+ (B) producing CD4+T cells per lung on different days after PR8/T4 infection in PBS and T4 preinfected mice. Data are mean ± s.e.m. from 5-11 mice in each group. PBS and T4 preinfected groups without asterisks are all not statistically different (NS). ****P < 0.0001; *P < 0.05. [file mbio.00519-23-s0003.tif]

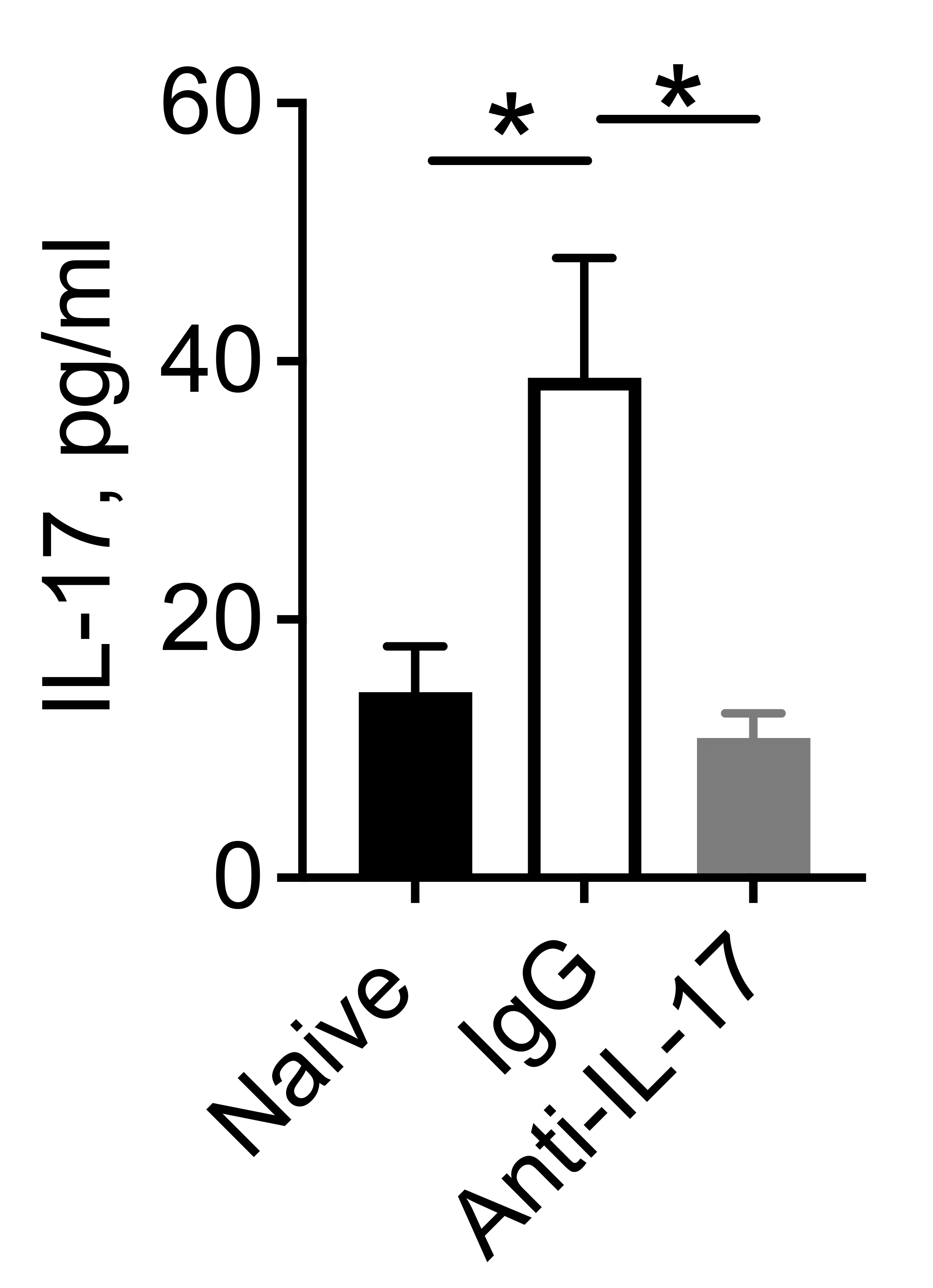

Supplement: Fig. S4 — IL-17 neutralization. T4-preinfected mice were first infected with PR8 and secondarily infected with T4. Preinfected mice were treated with anti-IL-17A antibody (anti-IL-17, clone 17F3) (n = 11) or the isotype control antibody (n = 10) intraperitoneally on days -1, 0 and 1 and intranasally on day 0 in comparison to naïve mice (n = 14) during T4 challenge following PR8 infection. The level of IL-17A in the BALF of mice was confirmed by ELISA 2 days after coinfection. Data are mean ± s.e.m.. *P < 0.05. [file mbio.00519-23-s0004.tif]

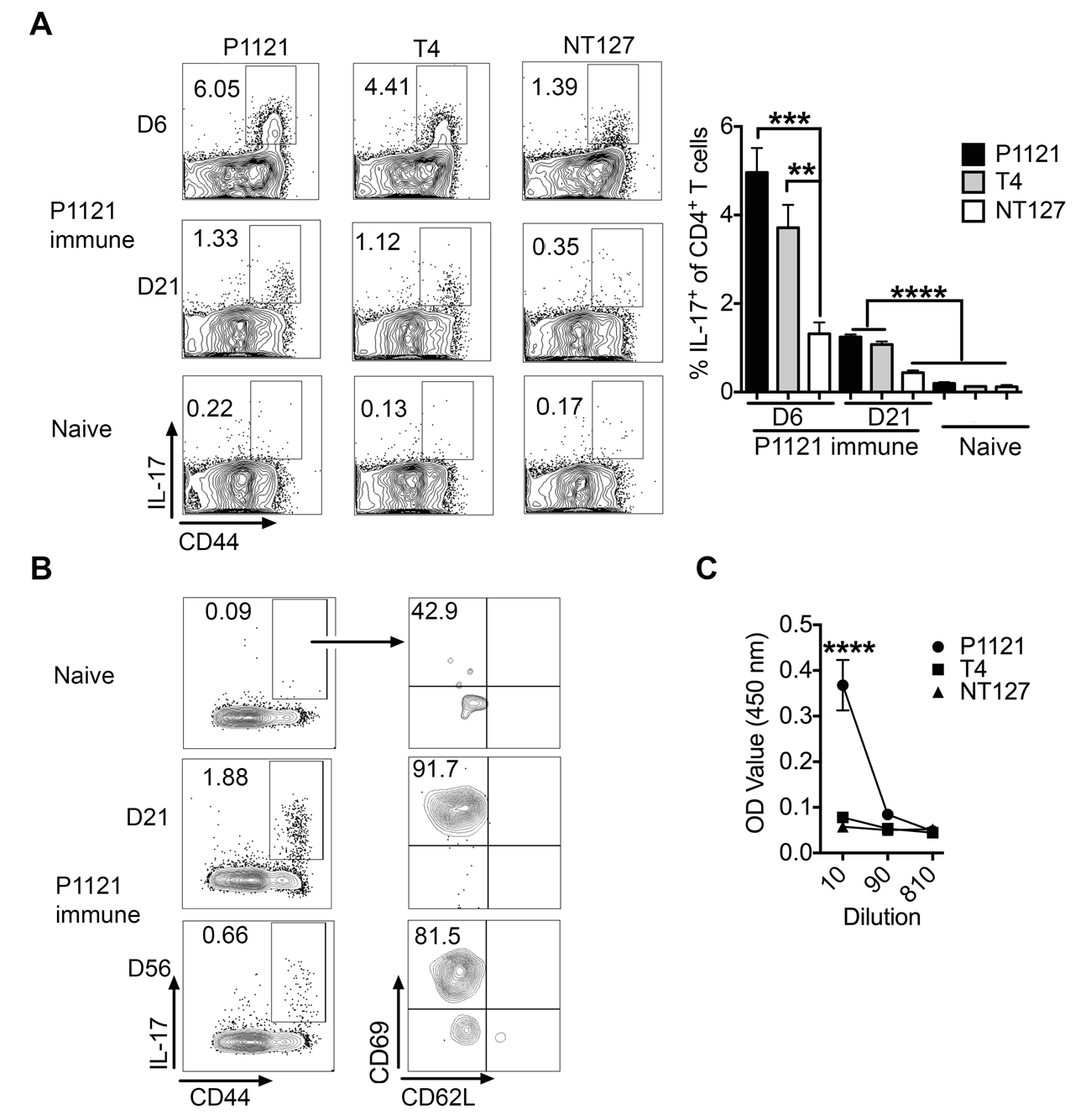

Supplement: Fig. S5 — Sp preinfection induces cross-reactive memory Th17 cells against different Sp serotypes. Percentage of IL-17A producing CD4+T cells after stimulation with heat-killed Sp (P1121 strain) and Sp (T4 strain) or H. influenzae (NT127 strain) on different days after P1121 exposure in P1121 infected mice or naïve mice (A). The phenotype of P1121-specific memory Th17 cells were examined in the lung on different days after P1121 infection according to their CD62L vs. CD69 expression (B). BALF immunoglobulin G (IgG) against different Sp (P1121 and T4) or NT127 from P1121 preinfected mice (C). Data are mean±s.e.m. from 5 mice in each group. ****P < 0.0001; ***P < 0.001; **P < 0.01. [file mbio.00519-23-s0005.tif]

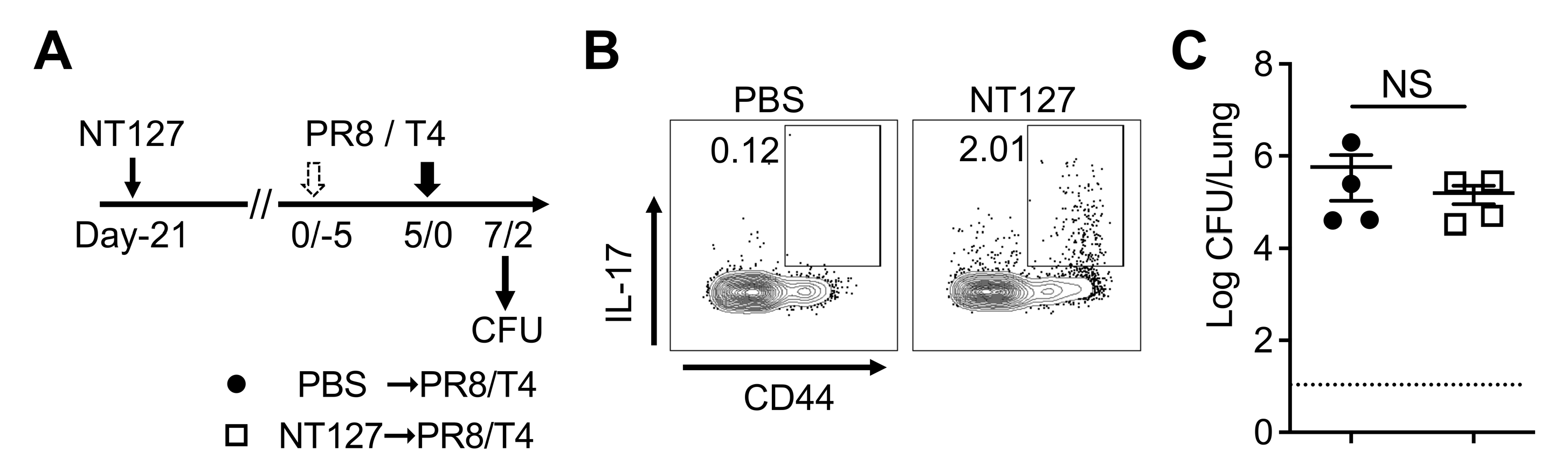

Supplement: Fig. S6 — Prior infection with H. influenzae does not provide protection against subsequent IAV/Sp coinfection. Mice were previously infected with H. influenzae (NT127 strain), and 21 days later challenged with PR8 followed by by Sp (T4 strain) infection (A). On day 21 post NT127 infection, IL-17 production by CD4+ T cells in the lung after stimulation with heat-killed NT127 was visualized by FACS (B). Bacterial loads in the lung on day 7/2 post PR8/T4 coinfection (C). Data are mean±s.e.m. from 4 mice in each group. NSP>0.05. [file mbio.00519-23-s0006.tif]
